# Positive Impact on Public Perception toward Commune Health Stations (CHSs) in Rural Areas of Tyuen Quang Province, Vietnam, Following the Application of the Development Program for the Capacity-Building of CHSs

**DOI:** 10.3390/ijerph20010754

**Published:** 2022-12-31

**Authors:** Yun Ju Jeong, Johny Bajgai, Jong Koo Kim, Dong Won Ahn, Young Jae Kim, Hyo-Jin Kim, Yundeok Kim, Cheol-Su Kim, Soo-Ki Kim, Kyu-Jae Lee

**Affiliations:** 1 Department of Convergence Medicine, Wonju College of Medicine, Yonsei University, Wonju 26426, Gangwon-do, Republic of Korea; 2 Department of Family Medicine, Wonju College of Medicine, Yonsei University, Wonju 26426, Gangwon-do, Republic of Korea; 3 Institute for Poverty Alleviation and International Development, Yonsei University, Mirae Campus, Wonju 26493, Gangwon-do, Republic of Korea; 4 Department of Internal Medicine, Division of Hematology-Oncology, Wonju Severance Christian Hospital, Wonju 26426, Gangwon-do, Republic of Korea; 5 Department of Microbiology, Research Institute of Metabolism and Inflammation Research, Wonju College of Medicine, Yonsei University, Wonju 26426, Gangwon-do, Republic of Korea

**Keywords:** medical services, communal health stations, outpatient satisfaction, Tuyen Quang, Vietnam

## Abstract

This study aims to discover whether or not the capacity-building intervention through implementing the “Rural Area Development Program” in Tuyen Quang province, in partnership with the Korea International Cooperation Agency (KOICA) and the Vietnamese Department of Health”, would positively affect the perception of the public toward the communal health stations (CHSs). To address this, three specific indicator-related satisfaction levels were examined regarding the infrastructure, the professional skills, and the service attitude of the medical personnel of the three CHSs toward outpatients. This cross-sectional study was conducted with 100 participants from three rural CHSs (Binh Yen, Vinh Loi, and Thang Long Communes). As a researcher-directed survey, a structured questionnaire was adopted to gauge the outpatient satisfaction levels in relation to the three indicators from the CHS medical milieu toward the patients and the medical services received. Descriptive and inferential analyses were performed to determine the perceptions of outpatient satisfaction relating to the three indicators. A higher satisfaction rate was found (overall 89–100% descriptive data with three indicators, as well as significant satisfaction differences in inferential data based on F-ratio and *p*-value) between the three regions with the three indicators, and two major data showed that the commune with a higher or more significant satisfaction rate or difference was Binh Yan > Vinh Loi > Thang Long. Collectively, this study clearly indicates the positive impact of CHSs capacity-building by implementing the Development Program in Tuyen Quang province with KOICA in relation to the public perception toward CHSs through significantly increased satisfaction levels—specifically, the infrastructure, the professional skills, and the service attitude of the medical milieu from the three CHSs toward outpatients.

## 1. Introduction

It is well known that sustainable development and public health are intricately connected. Achieving sustainable health development goals depends largely on the health outcomes of people [1,2]. Furthermore, a strong primary care system is required to provide an efficient and effective healthcare system [3]. Previous studies have reported that in many low- and middle-income countries, such as Vietnam, primary healthcare centers provide a wide range of services to people. The services provided include the identification and control of existing health-related problems, maternal and child healthcare, family planning measures, immunization facilities, emergency health services, treatment of common diseases, and the provision of essential drugs [3,4,5,6]. The primary healthcare centers additionally provide diagnostic and referral services to specialized hospitals. Despite the important activities of primary healthcare centers, there has been less focus on staff training. The implementation of such processes is needed to improve and update medical personnel’s knowledge, skills, and building of their capacity, especially in rural and remote settings, such as those in Vietnam [6].

Healthcare services in Vietnam are provided to people through tiered health systems at the central, provincial, district, and commune levels. Commune health stations (CHSs) work at the grassroots level to provide primary healthcare services to local populations. These stations are fundamental to the nation’s healthcare system and play a key role in implementing national health programs at the community level [7]. Additionally, the health government in Vietnam mobilizes CHSs to provide curative and preventive care facilities with services including family planning, immunization, and reproductive health services, as well as education programs related to health [8]. Vietnam currently contains more than 11,000 operational CHSs in 63 provinces across the country [9]. Furthermore, each CHS is equipped with a primary care physician, along with nurses, midwives, assistant doctors of traditional medicine, and pharmacists; these teams serve an average population of 8000 people in their catchment area [9]. Moreover, the quality of client experience with primary care in each CHS can be noticeably different, even within a setting in the same country. This variation remains a matter of concern for the CHSs. The difference in experience might be a result of several reasons such as (i) the quality of health services provided by less qualified CHS staff, as a result of inadequate professional knowledge, evaluative ability, and clinical skills; (ii) the limitations in the availability of drugs, equipment, infrastructure, and other clinical resources; and (iii) the geographical area, such as remote and mountainous regions, limiting access to and the quality of healthcare facilities [6,10].

To address and overcome these issues and gaps, numerous studies related to the capacity of healthcare services of CHSs have been conducted by the Vietnamese government, alongside foreign and domestic collaborators in Vietnam [8,11]. Patient satisfaction surveys are additional important indicators that can be used for determining the quality of healthcare services. It is, however, difficult to identify direct connections between patient satisfaction and the healthcare services provided. Implementing changes based on the feedback received is vital for upgrading the healthcare system and achieving optimal patient satisfaction [12,13]. Additionally, studies in Vietnam have reported that grassroot-level institutions, such as CHSs, have failed to attract people or gain their trust in making use of healthcare facility services. This reluctance by the public has led to increased crowding at higher-level facilities owing to the poor infrastructure, staff attitudes, equipment availability, and hygiene practices. Therefore, improving satisfaction among patients in CHSs will increase the utilization of local services, reduce overcrowding at higher-level facilities, and help policymakers identify the necessary improvements needed within the healthcare facilities [12,13,14].

To address the issue of public health, the Vietnamese health government also developed the Health Workforce Master Plan (2012–2020), which focuses on comprehensive measures to strengthen the quality of care at health centers in remote and mountainous areas [15]. This plan aimed to specifically increase the number of healthcare workers and improve the quality of care through training and education programs [15]. Other studies have, however, demonstrated that existing training and approaches for human resource development in CHSs are insufficient and further highlight the need for additional targeted training and developmental programs [6,11,16,17]. The “Rural Area Development Program in Tuyen Quang Province,” funded by the Korea International Cooperation Agency (KOICA), is to be implemented in Vietnam from 2019 to 2023, in partnership with the Tuyen Quang Department of Health. This program aims to increase the capacity for medical professionals working at communal and village levels, providing them with professional and technical training for the efficient and effective operation of healthcare services. Furthermore, the program focuses on the rural development components such as infrastructure (construction of CHSs), the socioeconomic empowerment of women, income generation activities, strengthening of public health services, education, and public administration. The rural development program in Tuyen Quang province conducted a survey in partnership with the KOICA and the Vietnamese Department of Health for outpatients in three CHSs (Binh Yen, Vinh Loi, and Thang Long) in 2022. In that context, the researchers propose to discover whether or not the capacity-building intervention through implementing the “Rural Area Development Program in Tuyen Quang Province, in partnership with the KOICA and Vietnamese Department of Health” would positively affect public perception toward CHSs. To address this, the researchers examined three specific indicator-related satisfaction levels, namely the infrastructure, the professional skills, and the service attitude of three CHS medical personnel toward the outpatients.

## 2. Materials and Methods

### 2.1. Study Design and Selection of Participants

This study was conducted as part of a feasibility study for the “Rural Area Development Program in Tuyen Quang Province,” funded by KOICA. The program will be implemented in Vietnam from 2019 to 2023, in partnership with the Tuyen Quang Department of Health. This cross-sectional study was conducted at three different CHSs (Binh Yen, Vinh Loi, and Thang Long) in rural parts of the Tuyen Quang province in Vietnam. 

The Tuyen Quang province spans 165 km and includes one city, six districts, and 129 communes, wards, as well as towns. This province is considered economically poor, ranking 48 out of 61 provinces in 2016. Additionally, most (86.3%) of the 667,214 residents of Tuyen Quang province live in rural areas. These regions have high unemployment rates, low infrastructure, and poor public health facilities. Currently, the “Rural Area Development Program in the Tuyen Quang province” project primarily covers the Son Duong and Ham Yen districts of Tuyen Quang province, including 17 communes (seven in Son Duong, four in Ham Ye, and six in other districts). The overall objective of the project was to inclusively and sustainably develop rural areas of the province in terms of infrastructure, women’s socioeconomic empowerment, income generation, education, public administration, and public health services (including CHSs). This study was conducted in three of these rural CHSs: two in the Son Duong district (Binh Yen and Vinh Loi communes) and one from the Ham Yen district (Thang Long commune). The objective was to strengthen public health services at a community level.

The researchers randomly recruited 300 outpatient participants in total (100 participants from each CHS) from March to June 2022. The researchers included data from participants older than 18 years of age. The data from the participants was collected by well-trained researchers. All participants were informed about the survey and provided their consent before the data were collected.

### 2.2. Data Collection Instruments and Measurements

For data collection, the researcher-directed survey was conducted with face-to-face interviews and adopted a structured questionnaire (Supplementary S1) that included questions relating to the respondents’ age, gender, and experiences within CHSs. The survey questions were divided into three indicators (Table 1) which were displayed as the shortened key inquiries in the next Tables of Results. The answers were recorded using a seven-point Likert scale [18]. As seen in Table 1, the first column as an indicator represents the outpatients’ satisfaction with the facilities of the newly constructed CHS building/facility, the second column represents the professional skills and service attitudes of the CHS medical personnel toward the patients, and the third column includes the medical services provided by the CHS, including health checkups, treatment, medicine prescriptions, maternal and pediatric care, population and family planning, as well as health education and communication. The participants were asked to evaluate the healthcare services provided by the CHS after the implementation of the “Rural Area Development Program in Tuyen Quang Province.” The participants also suggested further improvements relating to health services. 

Additionally, data obtained from the operational annual report (2018–2021) from the three CHSs (Vinh Loi, Binh Yen: December 2021; Thang Long: August 2021) over the four years were included.

### 2.3. Research Ethics

All participants were informed of the purpose and procedures of the study and voluntarily participated. For confidentiality purposes, the participants’ personal information was kept confidential and encrypted. 

### 2.4. Statistical Analysis

Before the questionnaire-based survey, to test the reliability of the question items, the researchers calculated Cronbach’s α as the reliability/internal consistency index. Descriptive and inferential statistics (ANOVA) were used to analyze the levels of patient satisfaction and to compare the patient satisfaction items between the three regions with the CHSs. The researchers summarized all of the findings using frequencies and percentages. All of the analysis conducted was performed using SPSS version 25.

## 3. Results

### 3.1. Respondents’ Participation and Baseline Characteristics

**Figure 1:**
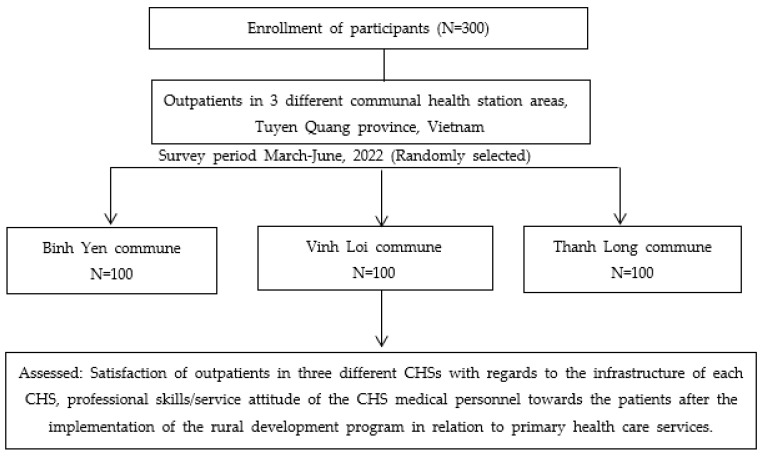
Flow chart diagram of the study design and participants for three commune areas in the Tuyen Quang province of Vietnam.

The data were collected from 300 participants across three different commune health station areas (Vinh Loi, Binh Yen, and Thang Long communes) in the Tuyen Quang province of Vietnam, as shown in Figure 1. 

The average age of the respondents in Binh Yen, Vinh Loi, and Thang Long communes was 36.43 ± 15.03, 46.74 ± 14.49, and 41.17 ± 17.21 years, respectively. Furthermore, the majority of the respondents were female, as shown in Table 2.

### 3.2. Reliability Index of Patient Satisfaction with the Three Indicators (CHS Building Facilities, Professional Skills, and the Service Attitudes of the CHS Medical Personnel, as Well as the Medical Services Provided at the CHSs)

To validate the reliability of this study’s questionnaire, the researchers calculated Cronbach’s (0.857) α as the reliability/internal consistency index which would validate the reliability of this study’s question items (Table 3). The researchers, therefore, conducted a satisfaction survey. 

### 3.3. Participants’ Satisfaction with the CHS Infrastructure

The participants’ satisfaction levels with the facilities of the CHS buildings are presented in Table 4. The data demonstrate that the majority of the respondents (97% in Binh Yen, 90% in Vinh Loi, and 81% in Thang Long) had “very good” satisfaction with the buildings. Similarly, most participants (97% in Binh Yen, 90% in Vinh Loi, and 85% in Thang Long) had “very good” satisfaction with the newly built CHSs, as compared to the old buildings/facilities.

### 3.4. Participants’ Satisfaction with the Professional Skills and Service Attitudes of the CHS Medical Personnel toward the Patients

The satisfaction levels of participants with the professional skills and service attitudes of CHS medical personnel toward patients are presented in Table 5. As demonstrated by the data, the majority of the respondents (97% in Binh Yen, 86% in Vinh Loi, and 75% in Thang Long) had “very good” satisfaction with the professional skills of the medical personnel at CHS. Similarly, the majority of respondents (97% in Binh Yen, 94% in Vinh Loi, and 75% in Thang Long) had “very good” satisfaction with the service attitudes of the CHS medical personnel in providing the medical examinations and treatment to the outpatients.

### 3.5. Participants’ Satisfaction with the Medical Services Provided at the CHSs 

Regarding participant satisfaction with the medical services provided by the three CHSs, the majority of participants (95% in Binh Yen, 90% in Vinh Loi, and 71% in Thang Long) had “very good” satisfaction with the medical services provided by the CHSs. Likewise, most respondents (96% in Binh Yen, 83% in Vinh Loi, and 74% in Thang Long) responded that they perceived an improvement in the medical facilities in the newly built CHSs, as compared to the old buildings. In addition, most respondents believed that the medical devices present were sufficient for the provision of services in the newly built CHSs (Table 6). 

Overall, the data from the survey indicates that the majority of people living in the Binh Yen commune were very satisfied with the newly constructed CHS building, its personnel, and the services provided, more so than residents of the Vinh Loi or Thang Long communes. In addition, the respondents from the Thang Long commune were the least satisfied with the CHS facilities.

### 3.6. Comparison of Patient Satisfaction Items between the Three Regions (CHS Building/Facilities, Professional Skills/Service Attitudes of CHS Medical Personnel, as Well as Medical Services Offered by CHSs)

To uncover the differences in satisfaction between the three healthcare centers, an ANOVA analysis was conducted. It was found that all seven satisfaction items were significantly different among the three regions at the 1% significance level (Table 7). In addition, Binh Yan showed a relatively higher average value (satisfaction inquiry item) as opposed to other regions, whereas Thang Long showed the lowest average value).

### 3.7. Healthcare Service Utilization across CHSs 

The utilization of healthcare services across the three different CHSs after the implementation of the “Rural Area Development Program in Tuyen Quang Province” is presented in Table 8. As demonstrated by the data, the highest availability of services and beneficiary population was found in Vinh Loi, followed by Thang Long, and Binh Yen (Table 8). Moreover, the highest rate of healthcare utilization by children under the age of five years old was also found in Vinh Loi, followed by Thang Long and Binh Yen (Table 8). Additionally, the researchers observed that a myriad of healthcare services was actively provided at all three CHSs, including outpatient and inpatient services, child delivery, the management of communicable diseases (such as AIDS, malaria, and non-infectious disease patients), and immunization services (Table 8). 

### 3.8. Availability of Basic Medical Device Equipment across the CHSs

The researchers found that all three CHSs had basic medical equipment (Supplementary S2).

## 4. Discussion

It has been established that patient satisfaction is an indicator of the quality of the healthcare provided. This indicator, however, is often overlooked and underemphasized [19]. This study clearly indicates the positive impact of the CHSs capacity-building by implementing the Development Program in Tuyen Quang province with the KOICA on the public’s perception toward the CHSs through significantly increased satisfaction levels—specifically concerning the infrastructure, professional skills, and service attitude from all three CHSs’ medical milieu toward the outpatients. Before the questionnaire-based survey, the researchers confirmed the high reliability of the seven questions asked by calculating Cronbach’s α (0.857) (Table 2). Based on this questionnaire, the researchers obtained meaningful results regarding the three indicator-linked satisfaction levels toward the CHSs. In this regard, the researchers speculate that the questionnaire could be a fair reference model for the study of rural CHSs in a similar milieu. 

This study’s positive results stem from two major pieces of evidence: a higher satisfaction rate (overall 89–100% descriptive data with three indicators; Table 4, Table 5 and Table 6), and significant satisfaction differences (inferential data based on the F-ratio and *p*-value) between the three regions and the three indicators (Table 7). Coincidently, two major data sets exhibited that the commune with a higher or more significant satisfaction rate or differences was Binh Yan > Vinh Loi > Thang Long. This coincidence might be ascribed to several factors, such as the medical accessibility and the different levels of the three indicators. However, this aspect remains to be elucidated. The significant differences in seven satisfaction items between the three health centers in Tuyen Quang province might be the first report relating to customer satisfaction following the capacity-building of CHSs in Vietnam. This has important implications, such as its application to other regions of Vietnam, and if this model proves successful, its extension to the similar milieu of other low- and middle-income countries. However, confirming the cause variable to explain the significant difference in satisfaction items between the three public health centers is warranted. In this regard, a follow-up survey is essential and still under planning. Furthermore, in parallel with the satisfaction survey, the observation of whether specific chronic diseases such as hypertension (HTN) and metabolic syndrome (MetS) will need to be controlled through cross-sectional or longitudinal studies is required. For example, there was an epidemiologic report on hypertension or diabetes in the communes of the same or different province [20,21,22]. 

The previous studies conducted on patient satisfaction with primary healthcare services also support the findings of this study [13,23]. Furthermore, studies have shown that in the last 20 years, the Vietnamese government has heavily invested in improving the quality of healthcare services at CHSs, by improving both the infrastructure and the quality of service provided by medical personnel at the grassroots level [3,24,25,26]. Moreover, a study of primary care centers in northern Vietnam reported that CHSs have a high potential for delivering prevention and treatment-related services [27]. As such, this study’s findings justify the investment in capacity-building for public health improvement in CHSs, suggesting that the CHSs are capable of providing effective, easily accessible, and comprehensive healthcare to the public, especially in rural settings. Similarly, a Chinese study found that CHSs provide primary care of equal or better quality than other healthcare service providers such as secondary- and tertiary-level hospitals [25]. This study’s findings affirm the CHS delivery model for providing grassroot-level care to the entire population, including the most marginal and vulnerable residents [28].

If outpatients who visited the CHSs were fully satisfied with the quality of their primary care, they were more likely to revisit the same facilities in the future. In this study, a large number of beneficiaries, such as patients, visited the CHSs from 2018 to 2021 for the utilization of healthcare services. This included services such as outpatient and inpatient services, child delivery, the management of communicable diseases, and immunization services. Additionally, the researchers found a high level of general service readiness for basic facilities, including the availability and functionality of basic amenities and medical equipment in all three CHSs after the program intervention. These results are also supported by previously published studies, which found that various government policies collaborating with international partners have since improved the infrastructure and equipment of grassroot health systems [8,29]. 

This study may hold pros and cons. First, there are no similar prior studies to enforce and support the public health improvement capabilities through official development assistance (ODA)—which is related to and inclusive of rural development programs in Vietnam. Previous studies were limited to the analysis of differences between health centers for education and regional health centers. This study, however, could cast insights into the analysis of improvements to public health in Vietnam’s rural areas, and the data could also be applied as a basis for the development of healthcare programs in a similar milieu in Vietnam or overseas. In contrast, the gender imbalance, representative of female-dominant participants in specifically the Binh Yen commune should be overcome for the objective reflection of the data from the Binh Yen participants.

## 5. Conclusions

Collectively, this study clearly indicates the positive impact of the CHSs capacity-building by implementing the Development Program in Tuyen Quang Province with KOICA on the public’s perception toward the CHSs through significantly increased satisfaction levels—specifically with regards to the infrastructure, the professional skills, and the service attitude from three CHSs’ medical milieu toward the outpatients. Furthermore, the allocation of adequate medical devices and training programs to medical personnel may be required to improve the medical services provided to rural populations. However, further in-depth studies are needed to better assess healthcare facilities after the completion of the program.

## Figures and Tables

**Table 1:** Key inquiry into the three participant satisfaction indicators for the medical milieu offered by the CHSs.

	Satisfaction Indicator	
Infrastructure (Building/facility)	Professional skills/service attitudes	Medical services
Satisfaction with newly built CHS (Building/facility)	Satisfaction with the professional skills of CHS medical personnel in providing medical examinations and treatment to outpatients (Professional skills)	Satisfaction with medical services provided in CHS (Medical services)
Satisfaction difference between new and old CHS building/facility (Comparison between building/facility; old and new)	Satisfaction with the service attitudes of CHS medical personnel in providing medical examinations and treatment to outpatients (Service attitudes)	Difference in medical services between new and old CHS (Comparison between medical services; old and new)
		Sufficiency of medical devices in CHS (Sufficiency of medical devices)

**Table 2:** Demographic characteristics of the research participants.

Characteristic	Binh Yen Commune	Vinh Loi Commune	Thang Long Commune
Age (In years)	36.43 ± 15.03	46.74 ± 14.49	41.17 ± 17.21
Gender (%)			
Male	28	44	39
Female	72	56	61

**Table 3:** Reliability index of patient satisfaction-relevant key inquiry with regards to the three indicators (CHS building/facilities, professional skills and service attitudes of the CHS medical personnel, as well as the medical services offered by CHSs).

Key Inquiry on the Satisfaction	Mean	S.D.	Corrected Item-Total Correlation	Cronbach’s α If Item Deleted	Cronbach’s α
Building/facility	2.913	0.283	0.606	0.840	0.857
Comparison between building/facility; old and new	2.899	0.302	0.659	0.833	
Professional skills	2.865	0.362	0.635	0.835	
Service attitudes	2.898	0.314	0.593	0.841	
Medical services	2.868	0.339	0.699	0.826	
Comparison between medical services; old and new	2.861	0.347	0.660	0.831	
Sufficiency of medical devices	2.848	0.453	0.580	0.852	

**Table 4:** Participants’ satisfaction with the CHS infrastructure (building/facilities).

Key Inquiry on Satisfaction	Response		Name of Commune	
		Binh Yen N (%)	Vinh Loi N (%)	Thang Long N (%)
Building/facility	Good Very Good	2 (2) 97 (97)	10 (10) 90 (90)	14 (14) 85 (85)
Comparison between the old and new building/facility	Good Very Good	2 (2) 97 (97)	11 (11) 89 (89)	17 (17) 81 (81)

CHS: commune health station.

**Table 5:** Participants’ satisfaction with professional skills/service attitudes of CHS medical personnel.

Key Inquiry on Satisfaction	Response		Name of Commune	
		Binh Yen N (%)	Vinh Loi N (%)	Thang Long N (%)
Professional skills	Fairly Good Good Very Good	0 (0) 1 (1) 97 (97)	0 (0) 13 (13) 86 (86)	2 (2) 22 (22) 75 (75)
Service attitudes	Fairly Good Good Very Good	0 (0) 1 (1) 97 (97)	0 (0) 5 (5) 94 (94)	1 (1) 22 (22) 75 (75)

CHS: commune health station.

**Table 6:** Participants’ satisfaction with the medical services offered by the CHSs.

Key Inquiry on Satisfaction	Response		Name of Commune	
		Binh Yen N (%)	Vinh Loi N (%)	Thang Long N (%)
Medical services	Good Very Good	3 (3) 95 (5)	10 (10) 90 (90)	26 (26) 71 (71)
Comparison between medical services; old and new	Good Very Good	2 (2) 96 (96)	17 (17) 83 (83)	22 (22) 74 (74)
Sufficiency of medical devices	Fairly Good Good Very Good	0 (0) 2 (2) 96 (96)	1 (1) 5 (5) 94 (94)	1 (1) 24 (24) 64 (64)

CHS: commune health station.

**Table 7:** Comparison of the patient satisfaction-relevant key inquiry between the three regions (CHS building/facilities, professional skills/service attitudes of the CHS medical personnel, as well as medical services provided at the CHSs).

Key Inquiry on Satisfaction		Binh Yeh	Vinh Loi	Thang Long	F-Ratio *p*-Value
Building/facility	Mean (S.D.)	2.980 (0.141)	2.900 (0.302)	2.859 (0.350)	4.825 0.009 *
Comparison between building/facility; old and new	Mean (S.D.)	2.980 (0.141)	2.890 (0.314)	2.827 (0.381)	6.661 0.001 *
Professional skills	Mean (S.D.)	2.990 (0.101)	2.869 (0.339)	2.734 (0.486)	12.975 0.000 *
Service attitudes	Mean (S.D.)	2.990 (0.101)	2.950 (0.220)	2.755 (0.455)	17.442 0.000 *
Medical services	Mean (S.D.)	2.969 (0.173)	2.900 (0.302)	2.732 (0.445)	13.708 0.000 *
Comparison between medical services; old and new	Mean (S.D.)	2.980 (0.142)	2.830 (0.378)	2.771 (0.423)	9.933 0.000 *
Sufficiency of medical devices	Mean (S.D.)	2.980 (0.142)	2.930 (0.293)	2.620 (0.677)	19.729 0.000 *

Note: ‘*’ represents significant difference at the 0.05 level.

**Table 8:** Healthcare services utilization at the three different CHSs in Tuyen Quang province, Vietnam from 2018–2021.

	Commune	2018	2019	2020	2021
Beneficiary population (N)	Vinh Loi	9109	9367	10,369	10,250
	Binh Yen	2980	3056	3137	3256
	Thang Long	7212	7266	7332	7360
No. of children under 5 (N)	Vinh Loi	1063	1004	1037	977
	Binh Yen	325	315	297	358
	Thang Long	690	606	589	518
No. of daily outpatient Total (Women, Children) (N)	Vinh Loi	32	25	18	15
	Binh Yen	30	26	55	70
	Thang Long	31	23	18	15
No. of inpatients per year (N)	Vinh Loi	45	33	15	18
	Binh Yen	36	48	32	58
	Thang Long	5	5	2	2
AIDS (N)	Vinh Loi	11	11	11	11
	Binh Yen	2	3	3	5
	Thang Long	1	1	1	1
Malaria (N)	Vinh Loi	0	0	0	0
	Binh Yen	1	1	0	0
	Thang Long	0	0	0	0
No. of delivery in CMS (N)	Vinh Loi	18	11	3	0
	Binh Yen	20	15	19	18
	Thang Long	5	5	2	2
Rate of wasting malnutrition of children under 5 (%)	Vinh Loi	15.8	15.74	15.7	15.69
	Binh Yen	18.6	18.2	17.8	17
	Thang Long	14.8	13.7	12	11.4
No. of non-infectious disease patients managed (N)	Vinh Loi	347	394	432	445
	Binh Yen	90	95	96	115
	Thang Long	106	127	166	204
Rate of children under 5 fully vaccinated (%)	Vinh Loi	99.6	99.2	99.4	99.7
	Binh Yen	96	98	98	99
	Thang Long	95	97	97	98
Average fertility reduction rate/year (%)	Vinh Loi	0.2	0.2	0.2	0.2
	Binh Yen	0.2	0.2	0.3	0.3
	Thang Long	1.27	1.15	1.55	0.96

## Data Availability

The data presented in this study are available in the article (tables and figures).

## Supplementary Materials

The following supporting information can be downloaded at: https://www.mdpi.com/article/10.3390/ijerph20010754/s1, Supplementary S1. Survey Sheet to Check on the Satisfaction of the New CHS and CHS Personnel Education Program. Supplementary S2. Availability of Basic Medical Equipment across CHSs.

## References

[1] Tuan T., Dung V.T., Neu I., Dibley M.J. (2005). Comparative quality of private and public health services in rural Vietnam. Health Policy Plan..

[2] World Health Organization Sustainable Development Goals, Goal 3: Ensure Healthy Lives and Promote Well-Being for All at All Ages. https://www.un.org/sustainabledevelopment/health/.

[3] Hoe N.T., Tam N.M., Derese A., Markuns J.F., Peersman W. (2019). Patient experiences of primary care quality amongst different types of health care facilities in central Vietnam. BMC Health Serv. Res..

[4] Toan N.V., Trong L.N., Höjer B., Persson L.A. (2002). Public health services use in a mountainous area, Vietnam: Implications for health for policy. Scand. J. Public Health.

[5] Chang C.H., Stukel T.A., Flood A.B., Goodman D.C. (2011). Primary care physician workforce and Medicare beneficiaries’ health outcomes. JAMA.

[6] Giang P.N., Kelly M., Nhung N.T.T., Sarma H. (2022). Continuing medical education programs for primary care physicians from remote locations of Vietnam: A needs assessment. BMC Med. Educ..

[7] Nguyen T.K., Cheng T.M. (2014). Vietnam’s health care system emphasizes prevention and pursues universal coverage. Health Aff. (Millwood).

[8] Tran Q.A., Le Tu H., Hoang Van M. (2021). Availability and readiness of communal health services: Results from 2015 Vietnam District and Commune Health Facility Survey. Int. J. Healthc. Manag..

[9] Nam V., Ministry of Health (2018). Population, Social, Economic and Environmental Indicators. Health Statistics [Yearbook].

[10] Kien V.D., Van Minh H., Giang K.B., Ng N., Nguyen V., Tuan L.T., Eriksson M. (2018). Views by health professionals on the responsiveness of commune health stations regarding non-communicable diseases in urban Hanoi, Vietnam: A qualitative study. BMC Health Serv. Res..

[11] Van Minh H., Do Y.K., Bautista M.A., Tuan Anh T. (2014). Describing the primary care system capacity for the prevention and management of non-communicable diseases in rural Vietnam. Int. J. Health Plan. Manag..

[12] Schommer J.C., Kucukarslan S.N. (1997). Measuring patient satisfaction with pharmaceutical services. Am. J. Health Syst. Pharm..

[13] Quyen B.T.T., Ha N.T., Van Minh H. (2021). Outpatient satisfaction with primary health care services in Vietnam: Multilevel analysis results from the Vietnam health facilities assessment 2015. Health Psychol. Open..

[14] (2015). Health Examination Administration-Ministry of Health Report of Medical Examination Management of the Year 2014 and Plan for 2015. https://vbpl.vn/pages/portal.aspx.

[15] Oanh T.T.M., Phuong N.K., Tuan K.A. Sustainability and Resilience in the Vietnamese Health System; Health Communications Strategy and Policy Institute: Vietnam, 2021. http://www3.weforum.org/docs/WEF_PHSSR_Vietnam_Report.pdf.Accessed.

[16] Vujicic M., Shengelia B., Alfano M., Thu H.B. (2011). Physician shortages in rural Vietnam: Using a labor market approach to inform policy. Soc. Sci. Med..

[17] Thu N.T., Wilson A., McDonald F. (2015). Motivation or demotivation of health workers providing maternal health services in rural areas in Vietnam: Findings from a mixed-methods study. Hum. Resour. Health.

[18] Satpathy S., Wundaville L.T., Satapathy S., Malik A., Singh S., Singh A.R., Chadda R., Barre V.P., Tiwari S.K. (2022). A systematic review of patient satisfaction scales and their applicability to COVID-19 hospitalized patients: Gaps and emerging needs. J. Patient Exp..

[19] Fenton J.J., Jerant A.F., Bertakis K.D., Franks P. (2012). The cost of satisfaction: A national study of patient satisfaction, health care utilization, expenditures, and mortality. Arch. Intern. Med..

[20] Bui Van N., Pham Van Q., Vo Hoang L., Bui Van T., Nguyen Hoang N., Do Nam K., Chu D.-T. (2018). Prevalence and Risk Factors of Hypertension in Two Communes in the Vietnam Northern Mountainous, 2017. BioMed Res. Int..

[21] Tran A., Khanh Tran T., Huong Phan D., Thi Ninh N. (2019). Prevalence of Metabolic Syndrome in Rural Areas of Vietnam: A Selected-Randomized Study. Arch. Pharma. Pract..

[22] Nguyen S.N., Tran V.D., Mai Le T.T., Nga H.T., Thi Tho N. (2021). High Prevalence of Metabolic Syndrome among Overweight Adults in Vietnam Based on Different Criteria: Results from a Community-Based Study. Clin. Epidemiol. Glob. Health.

[23] Schoenfelder T., Klewer J., Kugler J. (2011). Determinants of patient satisfaction: A study among 39 hospitals in an in-patient setting in Germany. Int. J. Qual. Health Care..

[24] Vietnam Ministry of Health (2016). Plan No. 139/KH-BYT Dated on 1 March 2016 Plan for People’s Health Protection, Care, and Promotion in the Period 2016–2020 (In Vietnamese). Ban Hành kế Hoạch bảo vệ, Chăm sóc và nâng Cao sức Khoẻ Nhân Dân Giai Đoạn 2016–2020. https://www.ncbi.nlm.nih.gov/pmc/articles/PMC6609979/.

[25] Vietnam Prime Minister (2016). Decision No. 2348/QD-TTg Dated on 5 December 2016 on Approval the Project of Building and Development Health Care Network at the Grassroots Level in the New Context (In Vietnamese). Quyết định phê duyệt đề án xây dựng và phát triển mạng lưới y tế cơ sở trong tình hình mới. https://lawnet.vn/en/vb/Decision-2348-QD-TTg-2016-development-of-the-grassroots-healthcare-network-in-the-new-context-67CF3.html.

[26] Vietnam Ministry of Health (2017). Circular No. 39/2017/TT-BYT Dated on 18 October 2017 on Health on Basic Package of Health Services Applied to Grass-Roots Health Facilities (In Vietnamese). Thông tư quy định gói dịch vụ y tế cơ bản cho tuyến y tế cơ sở. https://english.luatvietnam.vn/circular-no-39-2017-tt-byt-dated-october-18-2017-of-the-ministry-of-health-on-basic-package-of-health-services-applied-to-grassroots-health-facilit-117797-doc1.html.

[27] Duong D.B. (2015). Understanding the Service Availability for Non-communicable Disease Prevention and Control at Public Primary Care Centers in Northern Vietnam. Ph.D. Dissertation.

[28] Hu R., Liao Y., Du Z., Hao Y., Liang H., Shi L. (2016). Types of health care facilities and the quality of primary care: A study of characteristics and experiences of Chinese patients in Guangdong Province, China. BMC Health Serv. Res..

[29] Ministry of Health (VN) (2015). Joint Annual Health Review 2015 (jahr 2015): Strengthening Primary Health Care at the Grassroots Towards Universal Health Coverage.

