# Supplementary material for: Positive Impact on Public Perception toward Commune Health Stations (CHSs) in Rural Areas of Tyuen Quang Province, Vietnam, Following the Application of the Development Program for the Capacity-Building of CHSs
Source: Int J Environ Res Public Health. 2022 Dec 31;20(1):754. doi: 10.3390/ijerph20010754 (PMC9819934; doi:10.3390/ijerph20010754)
Supplement: Supplementary file 1 [file ijerph-20-00754-s001.zip › ijerph-2093987-supplementary.pdf]

International Journal of
Environmental Research and Public Health

MDPI

Supplementary S1. Survey Sheet to Check on the Satisfaction of the New CHS and CHS Personnel Education Program

Subject: 3 CHS personnel, patient/health (100/per CHS)
Questionnaire for better medical services in CHS to be provided in the future.

ICHS personnel/patient/health/checkup people		//						
Vinh Loi/Binh Yen/Thang Long		//						
Age/Sex/Date (month and day)		//						
Details		Likert scale 7						
**A. CHS building/facility**								
1	Are you satisfied with this new CHS building/facility?	−3	−2	−1	0	+1	+2	+3
2	How different is the new CHS building/facility compared to the old one?	−3	−2	−1	0	+1	+2	+3
3	Opinion and comment on the CHS building/facility							
**B. Medical device and services (only CHS personnel)**								
4	Are you satisfied with the new medical devices in the CHS?	−3	−2	−1	0	+1	+2	+3
5	How different are the new CHS devices compared to old ones?	−3	−2	−1	0	+1	+2	+3
6	Do you think that the medical devices provided are enough for medical services in CHS?	−3	−2	−1	0	+1	+2	+3
7	Do you think that the education program conducted in DOH is helpful with regard to conducting medical services in the CHS?	−3	−2	−1	0	+1	+2	+3
8	Opinions and comments on the medical devices							
**C. Skill and attitude of the CHS personnel (only patient and health checkup people)**								
9	Are you satisfied with the services and skills of the CHS personnel?	−3	−2	−1	0	+1	+2	+3
10	Are you satisfied with the attitude of the CHS personnel?	−3	−2	−1	0	+1	+2	+3
11	Opinions and comments on the medical services provided in the CHS							
**D. Medical services**								
12	Are you satisfied with the medical services in the CHS?	−3	−2	−1	0	+1	+2	+3
13	How different are the medical services provided by the new CHS compared to the old CHS?	−3	−2	−1	0	+1	+2	+3
14	Do you think that the medical devices provided are enough for efficient and effective medical services in the CHS?	−3	−2	−1	0	+1	+2	+3
15	Opinion and comment on the medical services in the CHS							
**E. Overview**								
16	1 medical service that satisfied you the most in the CHS							
17	1 issue that is needed the most in the CHS							

Note: *Likert scale 7: −3(very bad), −2(bad), −1(fairly bad), 0(same as before), +1(fairly good), +2(good), +3(very good).* Medical services include health checkups, treatment, medicine prescriptions according to health insurance, consultation and health care for mothers as well as children, the population, and family planning; and health education and communication.

Supplementary S2. Availability of Basic Medical Equipment across CHSs.

Medical Equipment Available at CHS	Vinh Loi Commune	Binh Yen Commune	Thang Long Commune
**I. General devices**			
Bed	P	P	P
Cabinet (next to bed)	P	P	P
Medicine cabinet with two chambers	P	P	P
Examination table	P	P	P
Examination light	P	P	P
Electronic blood pressure monitor	P	P	P
Nebulizer machine	P	P	P
Aspirator	P	P	P
Resuscitator (child + adult)	P	P	P
Oxygen generator	P	P	P
Minor surgery table	P	P	P
Minor surgery instrument kit (21 items)	P	P	P
Minor surgery instrument table	P	P	P
Emergency hand stretcher	P	P	P
Scale with height meter	P	P	P
Scale (child)	P	P	P
Stainless steel spatula	P	P	P
Stainless steel tray (825 mm, bean shape)	P	P	P
Stainless steel tray (475 mm, bean shape)	P	P	P
Flat instrument tray	P	P	P
Deep instrument tray	P	P	P
Gauche sterilizer (drum shaped, f24 cm)	P	P	P
Sterilizing box with lid	P	P	P
Infusion bottle holder	P	P	P
Clear headlight	P	P	P
Vaccine cold box	P	P	P
Oxygen tank + mask (child + adult)	P	P	P
Acupuncture machine	P	P	P
**II. Dental and Ophthalmology**			
ENT examination tool kit + head lamp	P	P	P
Tooth extraction kit	P	P	P
Ear forceps	P	P	P
Otoscope	P	P	P
Nose forceps	P	P	P
Nasal speculum	P	P	P
Eye forceps	P	P	P
**III. Examination devices**			
Urine tester (Germany)	P	P	P
Fridge	P	P	P
Glucose tester	P	P	P
Hematology analyzer	P	P	A
Biochemical testing machine	P	P	A
**IV. Obstetrics and Gynecology**			
Gynecological examination table	P	P	P

Obstetrics instrument table	P	P	P
Delivery tool kit (4 items)	P	P	P
Episiotomy kit (4 items)	P	P	P
Gynecological examination kit (3 items)	P	P	P
Pregnancy checkup kit (7 items)	P	P	P
Fetal heart monitor	P	P	P
Inox swivel chair	P	P	P
**V. Disinfectant equipment**			
Autoclave (steam pressure) 18 L	P	P	P
32 L dry heat sterilizer	P	P	P
Tongs	P	P	P

CHS, commune health station; P, present at CHS; A, absent at CHS.
